# RFID analysis of the complexity of cellular pathology workflow—An opportunity for digital pathology

**DOI:** 10.3389/fmed.2022.933933

**Published:** 2022-08-01

**Authors:** Lisa Browning, Kieron White, Darrin Siiankoski, Richard Colling, Derek Roskell, Eve Fryer, Helen Hemsworth, Sharon Roberts-Gant, Ruud Roelofsen, Jens Rittscher, Clare Verrill

**Affiliations:** ^1^Department of Cellular Pathology, Oxford University Hospitals NHS Trust, John Radcliffe Hospital, Oxford, United Kingdom; ^2^NIHR Oxford Biomedical Research Centre, Oxford University Hospitals NHS Foundation Trust, Oxford, United Kingdom; ^3^Nuffield Department of Surgical Sciences, John Radcliffe Hospital, University of Oxford, Oxford, United Kingdom; ^4^Philips Digital and Computational Pathology, Precision Diagnosis Solutions, Best, Netherlands; ^5^Department of Engineering Science, Institute of Biomedical Engineering, University of Oxford, Oxford, United Kingdom

**Keywords:** digital pathology, workflow, efficiency, time, analog, cost, radiofrequency identification (RFID), surgical pathology

## Abstract

Digital pathology (DP) offers potential for time efficiency gains over an analog workflow however, to date, evidence supporting this claim is relatively lacking. Studies available concentrate on specific workflow points such as diagnostic reporting time, rather than overall efficiencies in slide logistics that might be expected. This is in part a result of the complexity and variation in analog working, and the challenge therefore in capturing this. We have utilized RFID technology to conduct a novel study capturing the movement of diagnostic cases within the analog pathway in a large teaching hospital setting, thus providing benchmark data for potential efficiency gains with DP. This technology overcomes the need to manually record data items and has facilitated the capture of both the physical journey of a case and the time associated with relevant components of the analog pathway predicted to be redundant in the digital setting. RFID tracking of 1,173 surgical pathology cases and over 30 staff in an analog cellular pathology workflow illustrates the complexity of the physical movement of slides within the department, which impacts on case traceability within the system. Detailed analysis of over 400 case journeys highlights redundant periods created by batching of slides at workflow points, including potentially 2–3 h for a case to become available for reporting after release from the lab, and variable lag-times prior to collection for reporting, and provides an illustration of patterns of lab and pathologist working within the analog setting. This study supports the challenge in evidencing efficiency gains to be anticipated with DP in the context of the variation and complexity of the analog pathway, but also evidences the efficiency gains that may be expected through a greater understanding of patterns of working and movement of cases. Such data may benefit other departments building a business case for DP.

## Introduction

Digital pathology (DP) has taken center stage in the last few years within the setting of diagnostic pathology, and whilst to date only small numbers of laboratories worldwide have undergone a complete transition to DP for surgical pathology reporting, there are increasing numbers of departments embarking on the journey. The potential and actual benefits of adoption of DP are well-documented (1, 2); the promised utility of DP within diagnostic cellular pathology/surgical pathology services is many-fold (3), and includes the potential for time efficiency gains seen from a digital workflow as compared with the traditional “analog” workflow of glass slide microscopy. However, to date the evidence to support adoption of DP has necessarily been focused on proof of non-inferiority of digital diagnosis compared with traditional glass slides, with multiple large validation studies supporting the safety and quality aspects of DP for clinical diagnosis (4). Given the upfront financial investment needed to implement a DP set-up, the predicted cost-efficiencies associated with such gains become important within business cases put before funding bodies (3, 5, 6). Whilst generally it is claimed that DP is more time efficient and therefore has the potential to be more cost-efficient, much of the literature on this matter is subjective; there are very few published studies reporting on actual time efficiency or cost-benefit analyses of implementing DP and these parameters are difficult to capture and to compare due to inherent complexity of cellular pathology workflows, variability across centers and largely manual processes.

A report from a center in Granada, Spain (7) that has become fully digital, claims that since making the transition the pathologists are able to report on average 21% more cases per year. The detail of what facilitated this increase in productivity is not outlined in detail, but it would likely be related to more than just a change in the modality of reporting from glass to digital, and more a reflection of efficiency savings at multiple points within the journey of a case through the diagnostic laboratory. Few authors have attempted to break down the workflow pathway to analyze this in detail, and this is not surprising given its complexity. Ho et al. (8) sought to use contextual inquiry to gain an understanding of the complexities of the specimen journey, highlighting the concepts important to a pathologist within an analog workflow that would need to be considered during the development and transition to DP, in terms of how a pathologist approaches a case and progresses the case toward a diagnosis. This method was based upon observation of pathologists during a routine “sign-out service”, during which time notes were made of activities performed in order to construct affinity diagrams and graphical models of aspects of the work process, and served to illustrate inefficiencies in the analog workflow within the “pathologist role” including technical interruptions, deficiencies of data needed to complete a case, and inefficiencies of manual interpretation of diagnostic parameters such as mitotic counts. However, this study did not investigate the wider analog workflow.

Reports in the literature which detail the impact on laboratory/diagnostic workflow associated with the adoption of diagnostic DP are mainly descriptive texts around the “journey” to DP (7, 9–14). There are a few studies which include data on the impact of DP on pathologist reporting times (analog vs. DP) (15, 16) or more broadly on the “value-added” potential of digital pathology, by way of its impact on operational measures such as cost, time, service quality (17). Whilst some authors claim that actual diagnostic reporting time for a pathologist is equivalent or even reduced on the digital platform (15), a recently reported comprehensive equivalency and efficiency study set within the clinical workflow (16), concluded that DP was associated with a median overall 19% decrease in efficiency per case compared with glass. However, these studies looked only at reporting time for a case, rather than comparison of time within the entire workflow, and as acknowledged by the authors of the latter study, time savings elsewhere within the workflow may offset the apparent increase they demonstrated in turnaround time associated with digitally reporting a case, again underscoring the complexity of capturing what an efficiency saving is.

There are only a handful of studies which have been dedicated to analyzing and/or evidencing the differences that may be expected between analog (glass slide-based) and digital pathology pathologist working in terms of aspects of the diagnostic workflow (18–21). For example, the time in motion study (18) detailed the components of the pathway analyzed within the lab (case entry and case assembly time) and the separate analysis of the components undertaken by the pathologist, which were broken down into slide review, reporting (i.e., report writing), workflow-related, and other. This study highlighted the potential for a 13.4% saving in pathologist time related to workflow factors which would be expected to be negated in a digital setting. This figure of 13.4% potential time efficiency saving has been quite frequently quoted within the literature related to “benefits of DP” and has been translated by the same group to provide a figure for potential increase in pathologist productivity (19).

It is recognized that the analog workflow is inherently inefficient, with numerous “stop points” which would potentially disappear within a digital workstream, and these studies to date highlight at least some of these inefficiencies, although none with an overview of the entire relevant workflow. However, as finances within healthcare are increasingly scrutinized, further evidence around aspects of the analog workflow that will be altered or removed with the transition to DP, in a variety of laboratory settings, will be important for business cases going forward and this must be balanced against steps that will be added with DP such as cleaning of slides, loading slides into scanners and the time slides spend in scanners—waiting to be scanned, the scanning process (which in modern scanners is brief) and waiting to be unloaded.

We designed a novel study utilizing radiofrequency identification (RFID) technology to track the movement of surgical cases and personnel within a Cellular Pathology department over a set period of time, to analyze specific aspects of the workflow that we predicted could be at least partly negated in the move from an analog to a digital workflow. Through the unrestricted capture and analysis of both the physical movement of the slides and of personnel, and the duration of time for specific components of the workflow, we illustrate the complexity of the analog pathway and highlight areas of potential for efficiency savings and other significant gains in the digital workflow.

## Methods

### The study setting

The study was set in a large academic teaching hospital Cellular Pathology Department in 2019 prior to implementation of a fully digital pathology workflow for all surgical histology and referral cases which was completed in 2020 (13). The throughput is ~340,000 surgical histology and immunohistochemistry slides per year, together with 4,100 extra-large slides, and 40,000 referral slides. With 29 consultant histopathologists, 2 specialty doctors, and 9 trainees we operate a specialized service divided into 11 subspecialties, including pediatric pathology (but excluding neuropathology and soft tissue/bone pathology which are served within other laboratories). There is pathology support for 28 multidisciplinary team (MDT) meetings. The departmental footprint is that of a laboratory in one area, but with consultant and other offices geographically spread across the hospital site.

The study was designed to analyze the post-laboratory (pathologist focused) journey of the glass slide from the lab, to the pathologist for reporting, and eventually back to storage/filing, and the associated movement of personnel. The rationale behind this was the anticipated shift in slide logistics with DP; we hypothesized that this part of the glass slide journey would be effectively removed with a digital workflow, as most glass slides would then need only to go from the automated H&E stainer to the “sign-out” bench in the lab, to the scanner, and then to be stored/filed whereas the laboratory processes with the exception of slide scanning would remain largely unchanged.

The study was conducted as a service evaluation audit of current practice without the need for ethics committee approval. Signed consent was sought from personnel within the department for their participation in the study related to the use of anonymized data and potential photographic images.

### The RFID technology

Radiofrequency identification (RFID) technology uses electromagnetic fields to identify and track tags which can be embedded within an object. This negates the need for the object to be moved within the field of sight of a reader as would be necessary for a barcode, thus allowing the technology to be more discretely integrated with minimal impact on the system, in this case the laboratory workflow, being analyzed.

The use of RFID technology in healthcare is not new, with RFID technology deployed in a variety of settings such as the tracking of medical equipment/assets, tracking and managing drugs and patients, and tracking of blood supplies (22). Within pathology departments RFID technology has been utilized in recent years for quality improvement; to track pathology specimens and to ensure accurate patient identification (23–25). The technology for this study was therefore proven within the setting of a pathology laboratory, but within the literature to date had not been utilized specifically for analysis of time points within a pathology specimen workflow. The technology was selected for the ability to collect the complexity of data needed to track workflow in a cellular pathology laboratory.

### Pre-study planning

#### Analysis of the workflow

The first step in the study was to gain a clear understanding of the analog laboratory workflow. This was necessary in order to decide; (i) which aspects of the workflow would be tracked, and (ii) how these would be tracked in an effective way with minimal disruption to both workflow and staff in a busy department.

**Figure 1:**
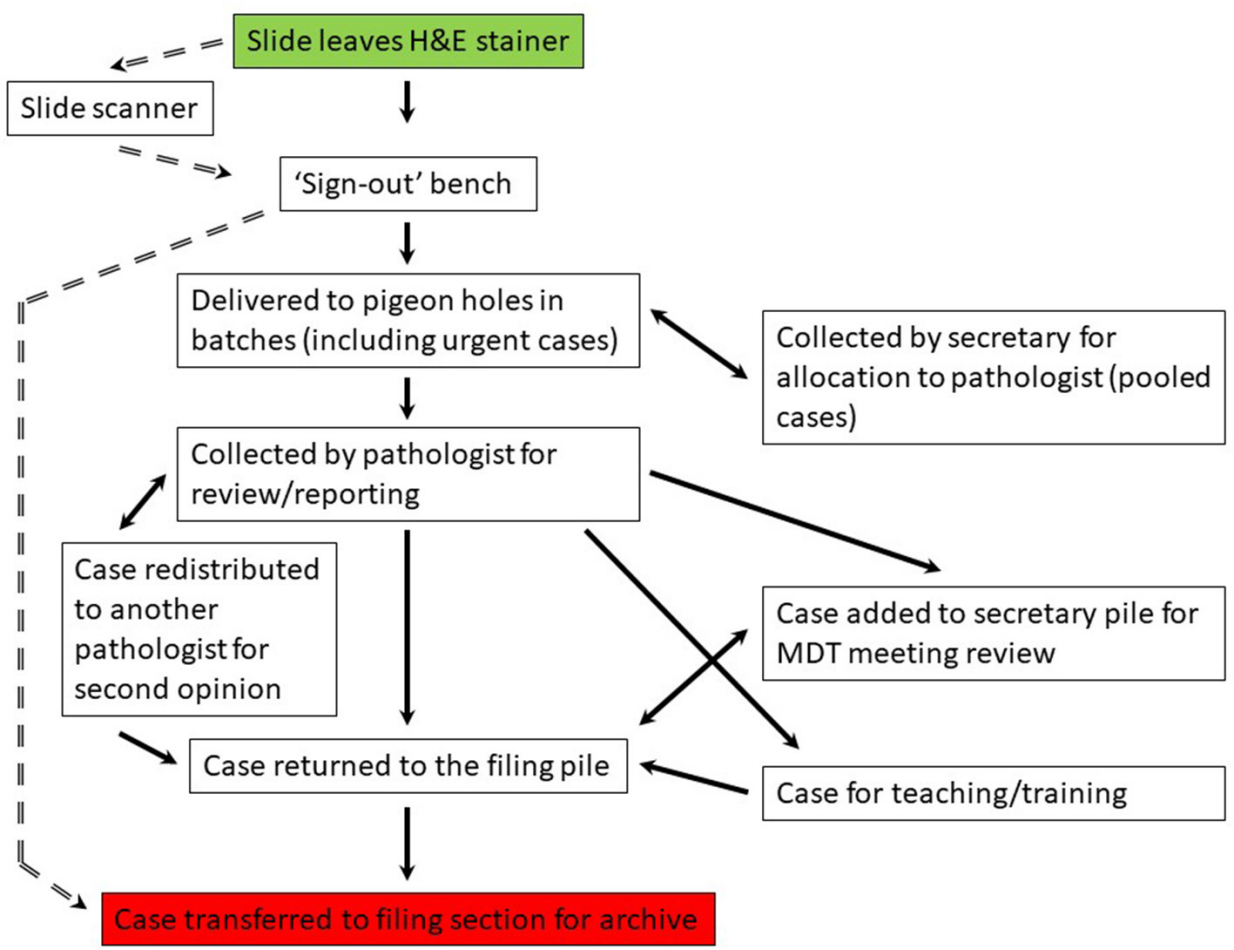
Process map for the laboratory workflow for a case from the point at which it is ready for pathology review to the return of the case for archiving following provision of a histology report. The analog workflow is on the right (solid arrows) and the predicted digital workflow is on the left (broken arrows). Start point (green) and end point (red) are the same for the analog and digital workflow. MDT, multidisciplinary team. Pigeon hole (PH) is the collection point for a diagnostic case by a pathologist, each having their own designated PH.

We undertook a process mapping exercise to detail the journey of a surgical case from the laboratory to the pathologist and back to filing following provision of a histology report, and in so doing were able to identify a start and end point which would be consistent within both the analog and digital workflow, with the other steps in the process predicted to effectively disappear following this transition (Figure 1). The study would track the components that would be most significantly impacted by the transition from analog to digital working, both in terms of movement of cases (“assets”) and personnel.

Optimal placement of the RFID readers was decided primarily on the basis of the workflow, although adjustments were necessary to minimize impact on the workflow itself, and to ensure technological success. Readers were placed at specific physical points which could be translated into an equivalent workflow point; at the start and end points of the workflow within the lab (the “sign-out” bench and “filing final”, respectively) and then at points which would register specific tasks; the pigeon holes (PH) from where pathologists collected their cases, the secretarial office to track movement related to case distribution for teams operating a “pooled” system of cases and for case movement related to multidisciplinary team (MDT) meetings, at pass points for pathologists as they relocated with the cases to their offices, the filing bench for return of reported cases by the PHs, and the final filing station within the lab from where slides would be archived. Slides are temporarily piled at the “filing pile” by the PH until they are relocated in batches by the laboratory technicians to the “filing final” bench within the lab from where they are returned to the archive.

#### The RFID equipment set-up

For the study we utilized a passive ultrahigh frequency (UHF) RFID system, with RFID tags for the tracking of cases, and RFID tags embedded in badges to be worn by personnel. Readers were installed for tracking of tags and for processing of the data and transmission to the data hub.

**Figure 2:**
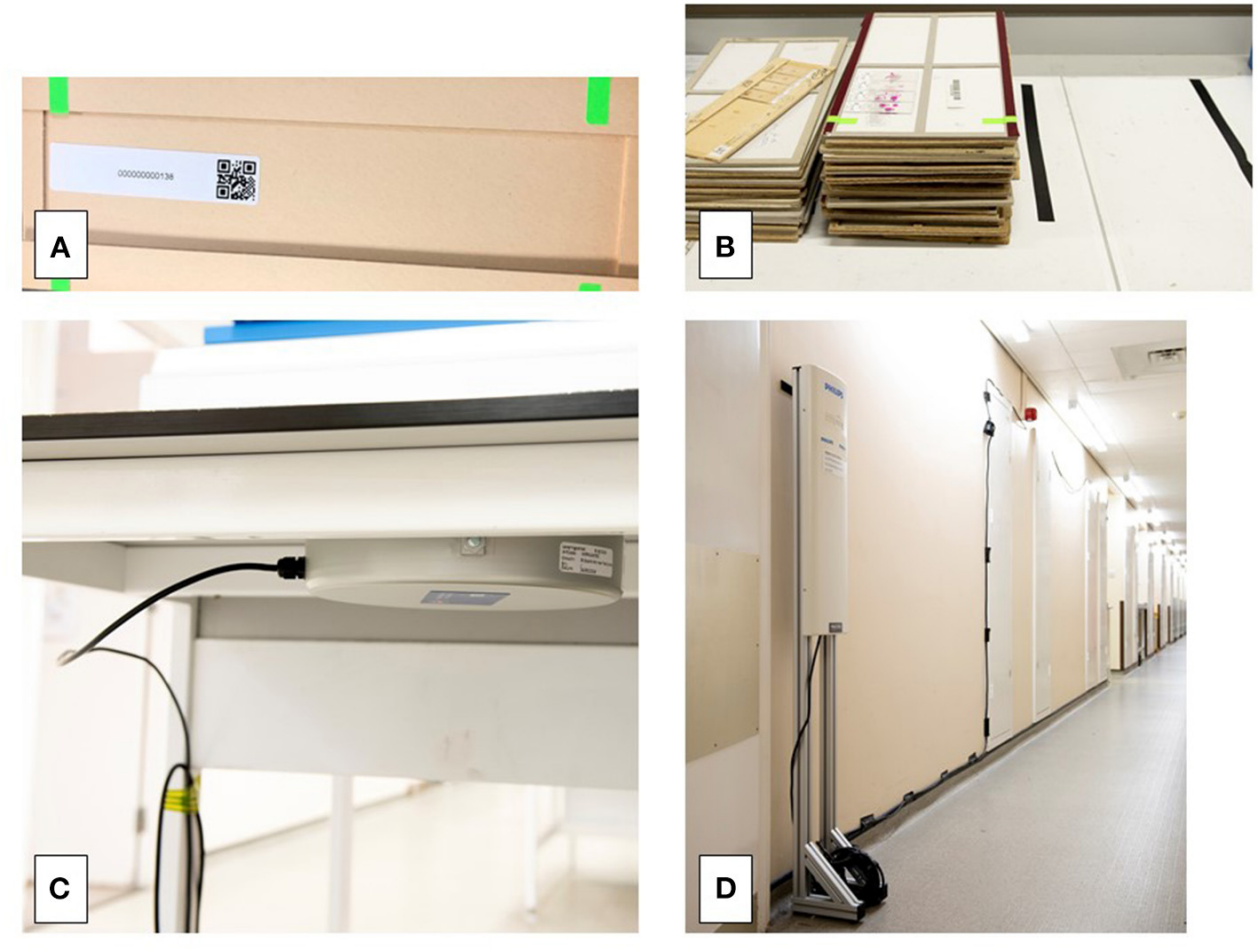
RFID tags attached to individual slide trays **(A)**, each tray designated for a single case. Tags could be detected by the RFID reader even when within piles of trays **(B)**. The position of the table top RFID reader (under the bench, see **C**) was indicated to the biomedical scientists by the placement of tape, within the bounds of which the tray was placed in order to “register” it **(D)**. This set up was present at the start and end point of the study at the sign-out bench and the filing final, respectively. Placement of RFID readers was on the basis of the process mapping of the workflow. The reader in D is that by the pigeon hole doors, placed to capture the movement of cases from the laboratory to the pigeon holes which was the point at which they were available to the pathologist. RFID, radiofrequency identification.

The RFID tags were individually attached to cardboard slide trays holding a surgical case—one case per tray in order to track movement of individual cases effectively. These trays were further identified with green labels in order that those handling the case (biomedical scientists, secretaries, pathologists) would be prompted to “register” the case on the desktop for specified fixed RFID tag readers (Figures 2A,B).

**Figure 3:**
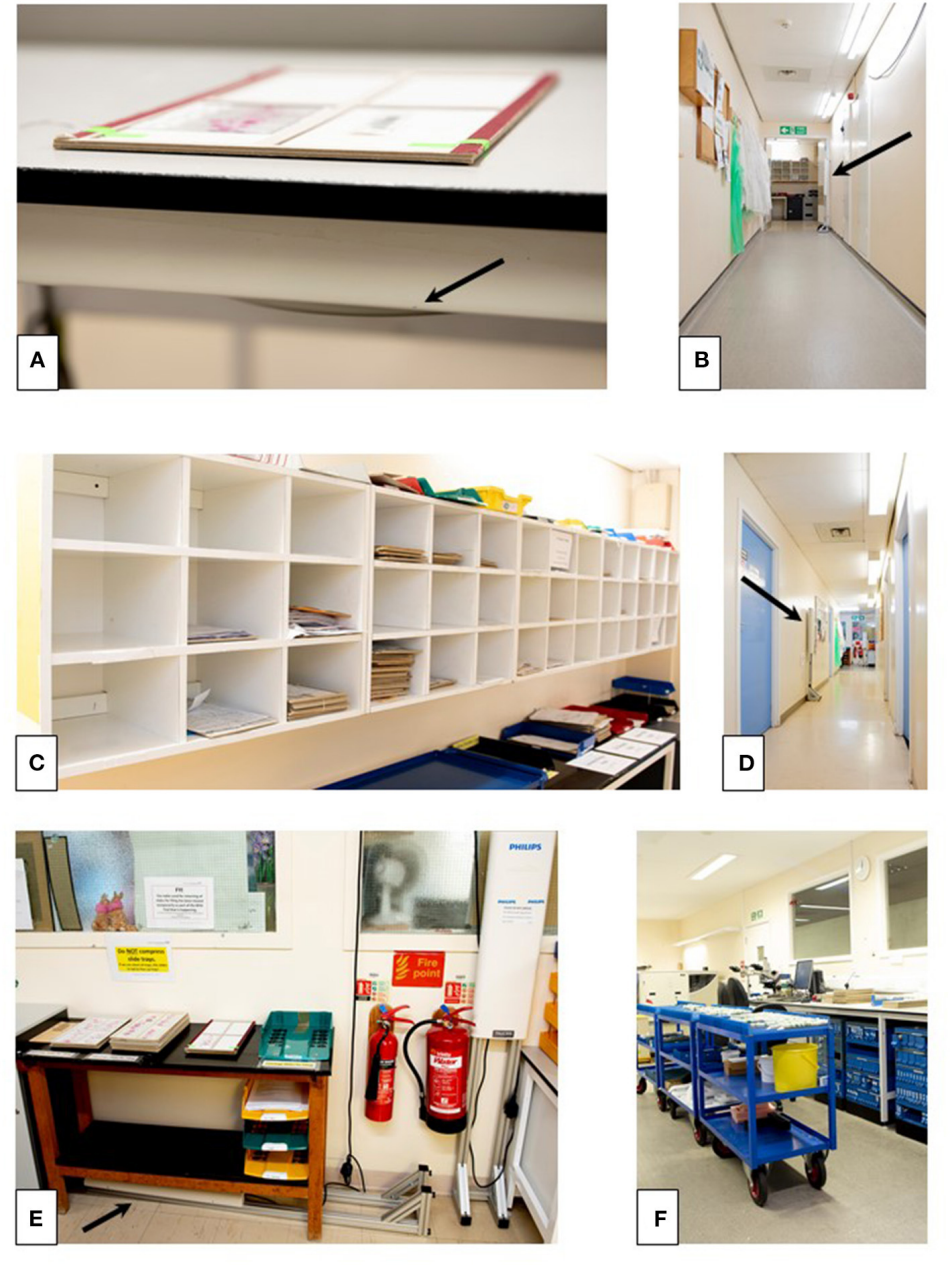
The “real-life” analog laboratory workflow which will effectively disappear with the transition to digital pathology. The cases leave the lab from the sign out bench **(A)** in batches, passing through the doors at the end of the lab (PH doors, **B**, see also Figure 2D) to be distributed into the PH **(C)** for collection by pathologists. Pathologists transport cases back to their workstations (offices) for review and reporting **(D)**, and then batch the cases prior to returning them to the filing bench (filing pile, **E**) from where they are collected several times daily and taken for filing at the “filing final” bench back in the laboratory **(F)**. The position of RFID readers in the images is indicated by the arrows. PH, pigeon holes.

The RFID readers were either free-standing (Impinj, Seattle WA USA, Speedway xPortal™ Integrated Portal Reader) to detect movement of a case or personnel, or designed to be placed under a desktop (Impinj, CS-777 Brickyard™ Near-Field Antenna) to read tags associated with slide trays/cases placed onto a worksurface (Figures 2C,D). Placement of the RFID readers was based upon the understanding of the workflow. A period of testing ensured operability of the system (tracking of cases and transmission of the data to the hub), and importantly it ensured that the system was acceptable to users with minimal impact on the process being analyzed, and that there was no interference from the RFID system on laboratory equipment, such as temperature monitoring devices for refrigerators/freezers. Readers were placed at the start point where cases leave the lab from the sign out bench (in batches), at the doors at the end of the lab through which cases pass before they are distributed into the PH for collection by pathologists (designated PH doors), at several pass points within corridors and doors through which it was predicted that pathologists pass whilst transporting cases back to their workstations (offices) for review and reporting, at the filing pile by the PH where reported cases are routinely returned and are then batched and transported to the final filing bench back in the laboratory (Figure 3). The critical placement of the readers to ensure that they detected tags on cases at a specific workflow point and were not detecting tags of cases at another workflow point, meant that in areas it was necessary to use surrogate locations for the readers (Supplementary Figure 1).

The RFID tags did not encode any patient identifiable data, and the RFID badges worn by personnel were anonymous; color-coded to the role of the wearer (lab technician vs. administrative staff vs. pathologist) and identified only by a number on the reverse.

The RFID data capture software (AUCXIS, Stekene Belgium, P-track) was installed on a laptop and this communicated with a database service (R-Connect). The RFID readers communicated over Wi-Fi with the R-Connect service through an access point. P-Track then visualized the raw data by means of a graphical user interface, providing the following output: (Time)—(EPC tag data)—(Tag Type) (Staff Badge or Tray Label) and (Reader Name) (location).

#### Case selection

We conducted the study over two separate time periods (study one = 21 days, 28 January to 17 February 2019, study two = 24 days, 4–27 March 2019) to allow sufficient time for most cases to follow the analog pathway (Figure 1), i.e., to leave the lab, undergo pathology review and reporting, and be returned to slide filing, allowing also for time for diversion from this pathway for example for MDT meeting review, second opinion, teaching.

The RFID labels were applied to slide trays for consecutive cases leaving the lab within the pathology specialties taking part in the study; urological pathology and gastrointestinal pathology (study 1), and breast pathology and dermatopathology (study 2). These specialties were selected due to; (i) the high-throughput of cases and turnaround times not impacted on for example by frequent requirements for ancillary tests associated with long lag-times such as molecular, and (ii) for specific workflow considerations such as the inclusion of the collation of cases in a pile in the secretarial office for the urology MDT meeting, or specialties operating a pooled system whereby biopsy cases were collected by a member of the secretarial staff once they had left the lab and then distributed evenly across pathologists within a specialty team, before being placed into the reporting pathologist's PH. These specialty-specific features would demonstrate the wider aspects of the analog workflow which would be transformed following transition to DP.

## Results

### Study 1—Gastrointestinal pathology and urological pathology case workflow

There were 695 tagged cases for this study period which included focus on two specific workflow aspects which will not exist in a digital workflow;

- For one specialty the movement of biopsy cases from a “pool” by a secretary from the collection point at the PHs to their desk (in batches) for redistribution of the cases amongst the consultant team, and then placement back in the relevant consultant PH.
- The collation of cases for an MDT (urology)—cases piled at a specific location in the secretarial office which would be included in the weekly MDT meeting list for discussion, and then returned after the MDT to the filing pile (and thence to filing final/archive).

**Figure 4:**
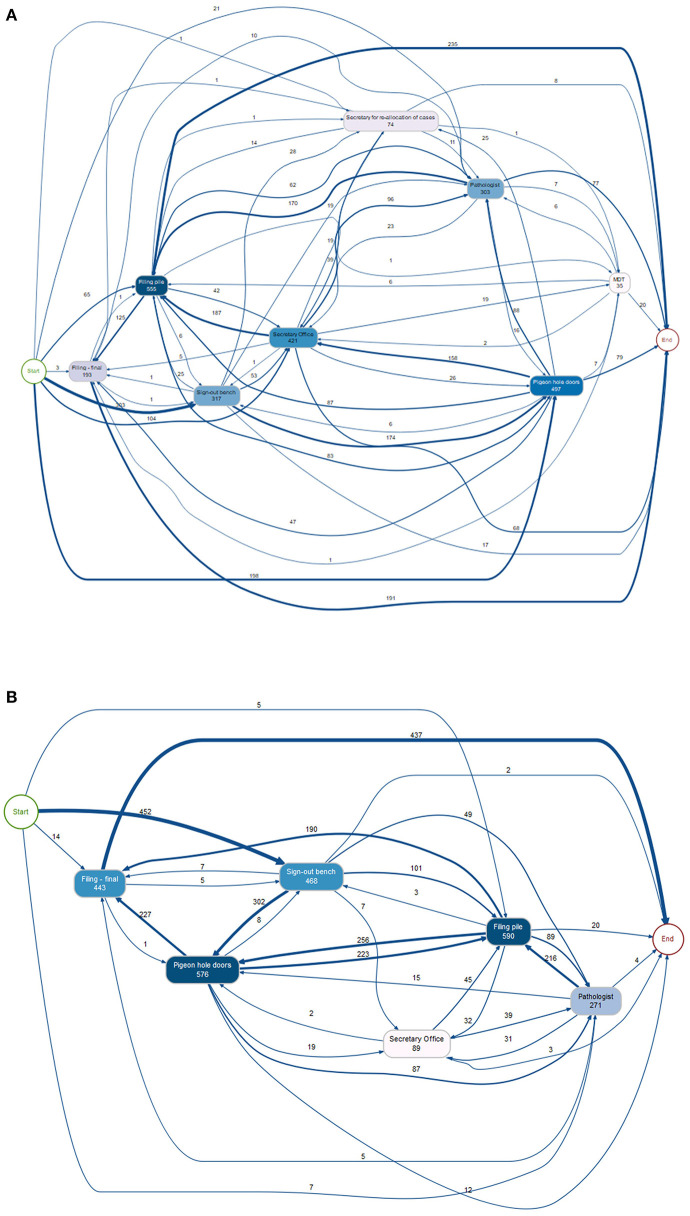
Diagrams showing the tracking of cases during the study period, with the number of cases following each pathway and being registered at each workflow point as shown, some being registered more than once at each point. **(A)** Study period 1 (695 cases). The movement of cases from the PH to the secretary for redistribution amongst a specialty team of pathologists, and then back to the PH for collection by a pathologist is shown (74 reads), as well as the movement of cases to the MDT pile for collation for the weekly meeting (35 reads), with 20 being returned to filing during the study period. **(B)** Study period 2 (478 cases). PH, pigeon holes; MDT, multidisciplinary team.

The trace data from this study period clearly demonstrates the complexity of the pathway that a diagnostic case takes from the time it leaves the lab (*via* the PH doors) for diagnostic reporting to the time at which it is returned to filing. Figure 4A shows the numbers of cases logged at various workflow points and the route of a case between these workflow points. Specific workflow points of interest were those associated with the “reallocation of cases” amongst a specialty team, and the movement of a case to and from a “pile” for an MDT, and there were 74 and 35 readings at these workflow points, respectively. The detailed analysis revealed that of the 695 tagged cases, 93 cases (13%) had been traced from the start of the analog journey at the sign-out bench in the lab to the end point at the “filing final” point, with 9% of the cases being traced from sign-out bench to “pathologist” to “filing final”. Whilst it is highly likely that a small proportion remained within the workflow at the end of the study period, particularly urological pathology cases which had been diverted to the pile of cases for the MDT meeting, it became apparent that technical tracking of tags was not optimal. This resulted in some traces not being complete and which was addressed prior to commencement of study period 2. The data from study 1 was then used to scope out the landscape of complexity of the pathway within the analog setting and inform subsequent analyses of the study 2 captured traces.

### Study 2—Breast pathology and dermatopathology case workflow

Following study 1 the positioning of the RFID detectors was revised to ensure better data capture. There were 478 traced cases for this second study period. As for study 1, the workflow points captured by the RFID tags are illustrative of the complexity of the journey of a case (Figure 4B). In fact, there were 113 unique trace patterns identified which recorded different patterns of physical movement of a case through the workflow. The most common trace pattern was sign out bench—PH doors—filing pile—filing final (59 traces). A proportion of the cases did not complete the analog “journey” during the study period, as would be expected, or showed traces with an illogical sequence such as filing final as the first timestamp. In total, 33 traces were completely excluded from further analysis. There were 445 traces remaining, however given that a timestamp had not been registered at each of the workflow points for all traces they were analyzed individually to determine which timestamps were available and thus which data could be analyzed from each trace. The breakdown of trace analysis is given in Supplementary Figure 2.

For the analyses, the timestamp at the PH doors was used as a surrogate for availability of a case in the pathologist's PH for reporting; batching of cases at the sign out bench following the quality check meant that this (sign out bench) timestamp was not a reliable indicator of readiness for reporting.

Timestamps have been analyzed to determine aspects of the analog pathway that will be significantly impacted by a transition to DP, principally around time taken for a case that is ready to leave the lab to actually be available for a pathologist to report, and the time that a case remains out of the laboratory within the workflow and therefore potentially untraceable, as follows:

1. Sign out bench to PH doors
2. PH doors to pathologist
3. Sign out bench to return of case to filing pile
4. Sign out bench to return of case to filing final
5. Pathologist to return of case to filing final
6. Filing pile to filing final

The time data for workflow points 1, 3, 4, and 6 is presented in Table 1, with the more detailed analyses to include the “pathologist” timepoints (points 2 and 5) presented separately below.

**Table 1:** Time taken between specified workflow points in the analog pathway.

	**Number of available traces with the data**	**Range of time (minutes)**	**Mean time (minutes)**	**Median time (minutes)**
Sign-out to PH doors	280	0.3–2,414.4	68.5	5.5
Sign-out to filing pile	387	3.6–29,014.6	5,855.8	3,912.8
Sign-out to filing final	402	214–27,047.6	7,134.9	4,395.3
Filing pile to filing final	396	1.2–20,185.7	1,461.8	1,069.2

PH, pigeon hole.

### Time taken for a case to become available for reporting (sign out bench to PH doors)

Once a case has been quality checked and is ready to leave the lab for reporting, it is batched at the sign out bench, and taken with other cases from the lab to be distributed within the PH. This activity is done in batches to make it time efficient within the analog pathway.

**Figure 5:**
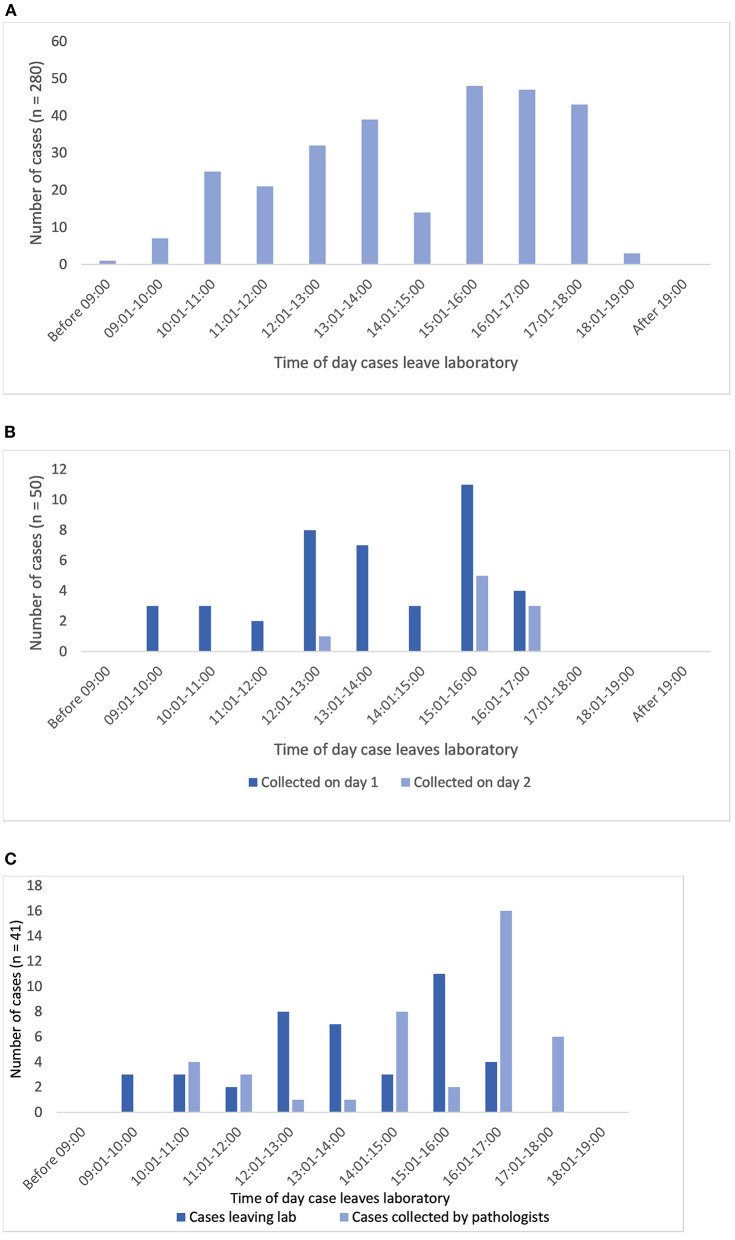
**(A)** Shows the distribution of times that diagnostic cases leave the lab *via* the pigeon hole doors at which point they are available for a pathologist to collect and report (*n* = 280 cases). **(B,C)** Show analysis limited to the traces with both PH doors and pathologist timestamps (*n* = 59 cases). **(B)** Shows the correlation of times that cases leave the lab and the times that pathologists collect cases (cases collected on day 1 or 2 only, *n* = 50). **(C)** Shows the time distribution of cases leaving the lab which were collected by pathologists on day 1 (same day, *n* = 41).

From Table 1 it can be seen that the median time a case would “wait” at the sign out bench was 5.5 min, and the mean time around an hour, however depending upon the time of day some cases would “wait” overnight to be delivered to the PH for reporting, with the longest delay being 40 h. Analysis of the timestamps for when cases were made available to pathologists (PH doors) is presented in Figure 5A. Whilst there is a spread of times that cases leave the lab during the day, it is seen that 45% of cases (125 of 280 with relevant traces) leave the lab before 14:00, although with few leaving the lab before 10:00, and that there is a peak between 15:00 and 18:00 when almost 50% of the cases leave the lab (138 of 280 cases). This is not unexpected given the pre-analytical laboratory steps for a case subsequent to routine overnight tissue processing (including block cutting, H&E staining, cover-slipping, slide labeling); tasks usually completed in the morning. It is noteworthy though that a third of cases left the lab after 16:00.

### Time taken for a case to reach a pathologist (PH doors to pathologist)

There were 59 traces with the pathologist timestamp in the workflow which provided data to illustrate when cases were collected for reporting, and a means to demonstrate patterns in the way in which a pathologist works within the analog pathway (see also Supplementary material).

Sixty-nine percent of the cases (41 of 59) were collected by a pathologist on the same day that they left the lab, and by the end of day 2, 85% of cases had been collected (50 of 59). The remaining 15% were collected on or after day 3, with the longest interval until collection of a case being 8 days. The lag time for the 15% of cases not collected on day 1 or 2 will include weekends and potentially other days a pathologist was not available, due for example to less than fulltime working.

In terms of length of time taken for the cases to be collected by a pathologist after leaving the lab (for 59 cases), the range was 0–9,594 min (day 8), with a mean of 1,199 min (~20 h) and a median of 125 min. Restricting the analysis to cases collected on day 1 (41 cases), the range was 0–1,320 min, with a mean of 118 min and a median of 79 min.

Figure 5B illustrates that there is a pattern to the time of day that pathologists appear to collect cases for reporting. Overall, of cases leaving the lab after 15:00 (*n* = 29), 15 (52%) are collected on day 1 but a further 8 (28%) are not collected until day 2, or later (20%). For cases collected on the same day as they have been made available from the lab (day 1), the peak time for cases to be collected by pathologists is between 14:00 and 18:00 (32 cases, 78%), with 22 cases (54%) being collected after 16:00 (Figure 5C). This appears to indicate that generally there is a lag of a couple of hours between the peak time at which cases are made available, and the time at which cases are collected. If this pattern holds true across all of the cases leaving the lab, then given that we have seen that a third of cases leave the lab after 16:00 it could be estimated that overall around 15% of cases per day will not be collected for diagnostic reporting until the following day if they are not “ready” from the lab for collection in the PH by 16:00.

### Time that a case remains out of the laboratory within the diagnostic workflow (sign out bench to return to filing)

The analysis of time that a case has spent in different parts of the workflow provides additional detail in relation to time that a case is effectively “out of circulation”; periods during which the case would potentially be difficult to locate within the department should it be needed.

**Figure 6:**
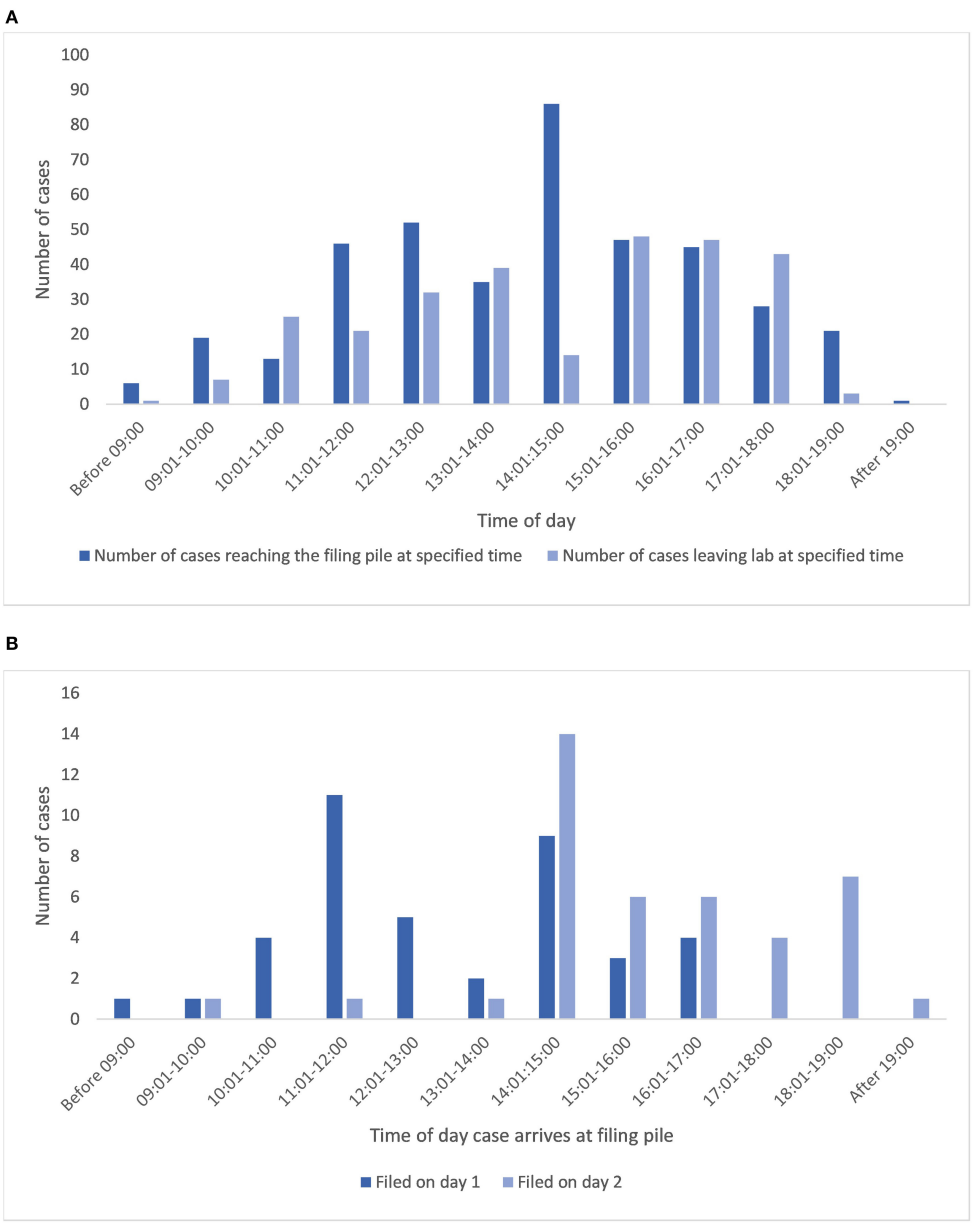
**(A)** Illustrates the times that cases leave the lab to become available for collection from the PH for reporting alongside the times that cases are returned to the lab for filing. **(B)** Shows the correlation between time of day a case arrives for filing, and the day that it reaches filing final, for cases filed on day 1 (same day) or 2 (data presented only for those cases with a pathologist in the trace). PH, pigeon hole.

From analysis of the relevant trace data (Supplementary Figure 2) it is seen that once a case has left the lab (sign out bench), the average time before it is returned to the final filing point for archiving is 7,135 min (almost 5 days), with the shortest period being 214 min, and the longest period recorded being 27,048 min (almost 19 days, Table 1). This time period partly reflects turnaround time for a case for generation of a diagnostic report, and we have not specifically collected data on this for the purpose of this study, however it will also include time periods for which the cases have been diverted from the pathway for an MDT meeting, or for activities such as teaching. It is recognized that pathologists also tend to batch cases for return to limit the number of trips to the lab, and indeed the times of day that cases are logged back at the filing pile for archiving show similarity to the times at which cases are made available for collection by pathologists (Figure 6A) indicating perhaps an attempt by pathologists to optimize time efficiency by returning reported cases when new cases are collected.

### Time taken for a case to reach the final filing station after it is returned to the lab

The onward return of the cases to filing final for archiving is an additional workflow inefficiency, as illustrated by the average wait of cases for 1,462 min (24 h) to be filed ready for archiving, although a good proportion of the cases were filed much faster than this (1st quartile = 114 min, data for 399 cases). The efficiency of this activity appears to be at least partly dependent upon the time that a case is returned for filing. Figure 6B illustrates the data related to this activity for traces which also included a pathologist and that were filed on day 1 or 2 after being returned, showing that 60% (24 of 40) of cases that reached the filing pile before 14:00 and 83% of those before 15:00 were filed (filing final) on the same day that they had reached the filing pile, whereas of the 41 cases filed on day 2, 93% had reached the filing pile after 14:00. Overall of these cases, 44% of cases were returned to the final filing/archive bench on the same day as they were placed in the filing pile, and 90% had been returned to filing final by the end of day 2, with 98% of cases having been filed by the end of day 4, and the rest by day 6.

### Movement of personnel

**Figure 7:**
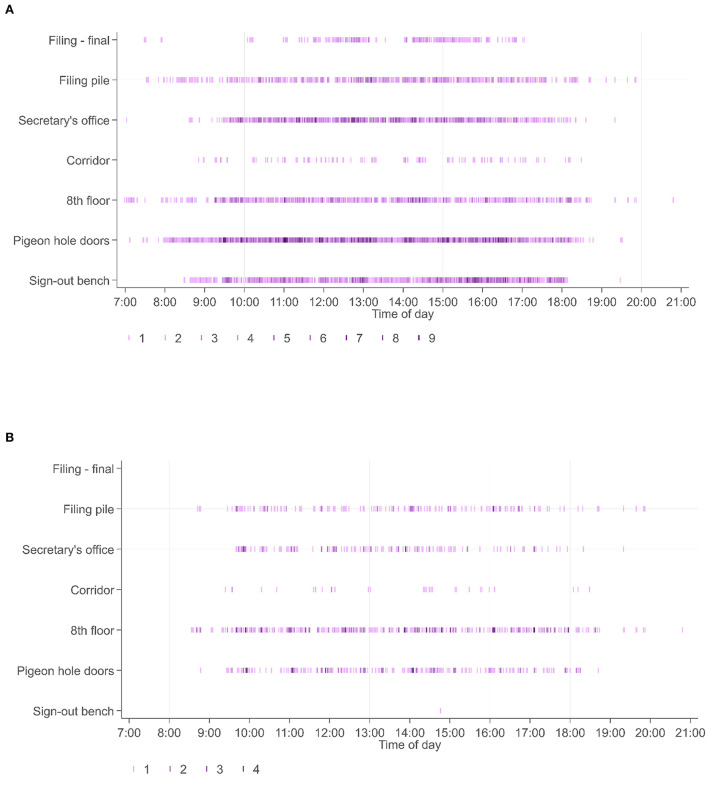
**(A)** Shows the timestamps at specific workflow points across all 31 personnel wearing RFID tagged badges (biomedical scientists, secretaries, pathologists), presented across the entire period for study 2. By contrast **(B)** shows the locations of pathologists within this workflow over the same time period. It is seen that pathologists do not spend time at the sign out bench or filing final pile (locations more frequently visited by biomedical scientists), and that the timestamps are related to readers associated with collection of cases (PH doors), relocation of cases to their offices (8th floor and corridor readers) or to return of cases (filing pile).

Movement of cases within the workflow was associated with movement of personnel, divided into the role categories of biomedical scientists, secretarial staff, and pathologists. For illustrative purposes we captured the movement of staff utilizing the RFID tagged badges they wore, detected at the same workflow points as the diagnostic cases. For study 2, 31 personnel wore RFID-tagged badges: 15 BMSs, 9 pathologists, 7 secretarial/administrative staff. Whilst the movement of individual personnel could be captured as they were registered at the various workflow points, the purpose of this part of the study was more to capture an overview of personnel movement; where different categories of personnel were at different times. This data (Figure 7), shows patterns in terms of the times of day that personnel are detected at various workflow points, with the PH doors (pigeonholes) being a particularly “busy” location, and patterns as to which locations people in particular roles are detected.

## Discussion

Considerable evidence exists within the literature around the pathologist experience and quality assurance aspects of DP, mostly around the validation process and concordance between DP and glass. However, in spite of claims that DP is of potential benefit in improvement of laboratory and pathologist efficiency there is a dearth of evidence around this subject, and specifically in respect to the potential impact of DP on laboratory workflow and slide logistics. In fact, a recent review by Jahn et al. (2) comments that “there is no real-life cost-efficiency analysis for full DP implementation with sufficient (>5 years) follow-up”. Most studies to date focus on operational savings as a whole rather than detailed analysis of existing workflows and the potential thereby to understand the elements that may benefit most from the transition to DP, not only through time efficiency gains, but in the wider context of patient safety and quality improvement. Baidoshvili et al. (20) undertook physical measurements of time taken within various aspects of an analog workflow, comparing this with a digital workflow in a laboratory setting in The Netherlands handling around 220 cases/day. They captured potential time efficiency savings in slide logistics of up to 1,147 min/day across five key workflows (>19 h), including case assembly and transfer to pathologists, slide archiving, MDT preparation. Their study provides an assessment of potential time efficiencies in components of the analog pathway, however it does not capture the real-life movement of cases within the analog workflow and the variability in this, nor the sheer complexity of the case journey, which has additional impact on routine working. It is the inherent difficulty in capturing the complexity of workflows that likely contributes to the lack of such available data, which we attempt to address in this novel study. Utilization of RFID technology has allowed automated and continuous tracking of cases throughout the entirety of the post-laboratory workflow, evidencing the actual movement of cases within the laboratory and wider department, and illustrating the pathways a case follows which are sometimes deviant from that expected, impacting potentially on case traceability. This technology has facilitated direct measurement of timestamps for cases at pivotal pathway points, allowing detailed observation of the analog workflow in a manner that is not easily captured with manual recording, although it was not issue free and adjustments were needed to capture the data more optimally.

A key aspect of the transition to DP often focused upon is the prediction of improved turnaround times (TAT) in diagnostic reporting. TAT is seen as a measure of quality within the practice of cellular pathology, which is influenced by a myriad of factors of which the laboratory need to be aware (26). Instinctively a shortened turnaround time would result from the instant availability of a case digitally after booking out from the lab, negating the need for the physical movement of a case to a pathologist, which in our department involves collection of cases from a designated location next to the lab, the “pigeon hole” (PH). Both the booking out of the cases from the lab at the sign out bench and the manual transportation of cases to the PH is typically done in batches, immediately conferring a delay in case availability. Our study has captured a mean lag time of around an hour for cases waiting in the lab to be taken to PHs, and a further lag time of around 2 h before pathologists collect cases from their PH for reporting. In our system therefore a digital workflow could potentially negate an average 2–3 h of “delay” in a case reaching a pathologist for reporting. Whilst there is accepted variability between laboratories in terms of workflow, this specific aspect of physically getting cases from the lab to the pathologist will be reproducible across laboratory settings to at least some extent and this data therefore offers an indication of a relevant efficiency gain that will be transferable. The study from Baidoshvili et al. (20) showed that similar workflow components (preparation of a case to leave the lab, batching, and transfer of cases to pathologist) took 640 min/day (for around 200 cases), and predicted that in the digital workflow this would be reduced to 36 min/day for the same number of cases. We also noted that whilst 52% of cases leaving the lab after 15:00 were still collected on the same day, a further 28% were not collected until the following day, or later (20%). Perhaps significantly, given that we demonstrated that around one third of cases leave the lab after 16:00, this potentially translates to 15% of cases not being collected for reporting on the same day that they leave the lab. These few hours “saved” through the instant digital access to a case could facilitate same day reporting of greater numbers of cases, or requests to the lab for extra work, also having potential impact on case turnaround time which is increasingly important as a performance indicator in addition to the direct benefit on patient management pathways. Furthermore, whilst not captured in detail in our study, the time taken for pathologists to physically collect and return cases and the interruptions in the working day needed to do this, will be an additional important efficiency saving with DP. The concept of the pathologist as an operator or machine who can only undertake one task at one time is described in a previous study of workflow scheduling in pathology laboratories (27).

In all of these assumptions it must be acknowledged that these potential savings will be dependent upon the set-up/order of workflow in a digital pathway. Furthermore, it should also be considered that in the overall TAT for a case within the workflow (from receipt to authorization of a diagnostic report), there will be an impact from the scanning time *per se*, and the loading of cases onto the scanner, as well as the quality control process (which is likely to vary between labs in terms of where it falls within the pathway). However, by contributing an understanding of the real-life journey of a case and the working patterns of both the lab and the pathologist within the existing analog workflow, this study may aide the design of a digital workflow to optimize efficiency gains.

The physical delivery of glass slide cases in batches for a pathologist to report, the “push” approach, does not generally allow ready identification of cases within the pile according to their clinical urgency nor does it accommodate variation in pathologist availability (28). We have shown there is a variation in lag time from the earliest opportunity that a case is available from the lab to the time it is collected for reporting, which includes extended periods likely accounted for by flexible working patterns. Indeed, whilst there was a median time interval of around 2 h (mean time interval of 20 h) before a case was collected for reporting we saw that 15% of cases were collected on day 3 or later after being made available from the lab. We do not have specific details as to working patterns to account for these findings, but it illustrates the variation that exists. Pooling of cases for reallocation across a team is an option available in an analog workflow to accommodate such variation, however this introduces additional manual work into the system such as secretarial redistribution with associated additional movement of cases as we have shown in the first part of our study. A digital workflow offers the opportunity for any pathologist to see all cases that are ready for reporting, which can be tagged according to clinical urgency to enable prioritization of reporting. Cases can be “pulled” from this digital workflow and more easily distributed amongst a team and in accordance with availability, potentially offering a ready means to smooth the flow of work and turnaround times.

Perhaps the most compelling feature of the data presented is the complexity of the pathways that these diagnostic cases took. Whilst we had process-mapped the predicted pathways we had not anticipated the extent and variation of the physical movement of a case around the department between the time it was available for reporting and the time that it was returned to the final filing point from which it could be safely archived. Significantly, the time period that a case is out of the lab but not in filing, is one in which it is typically challenging to locate in current analog systems, which results in significant wasted time looking for slides if they are needed, and the inherent risk of a case going “missing”. Whilst a case may be traceable when physically with a pathologist, we have shown that the cases spend significant redundant time at multiple other points within the pathway such as re-distribution by administrative personnel, in a pile for an MDT meeting, in a pre-archive filing pile, during which time the case is effectively out of circulation and would be difficult to locate if needed. In our study the average time that a case spent effectively “out of circulation” within the workflow (from sign out to return to filing final for archiving) was around 5 days, although the range was 214 min to 19 days. Part of this time was accounted for by a case waiting for an average of 24 h within a “pre-filing” pile to be transported to the filing station for archiving. By returning the glass slides to the archive after scanning, these remain accessible should they be needed, with the digital images freely accessible to those with access, whether this is for diagnostic reporting, teaching, MDT meetings, clinical trial review, etc.

Whilst we did not capture time taken by administrative staff to collate cases for an MDT meeting, as others have shown (20) this is also a point of potential time efficiency gain in the digital workflow, partly given that cases can evidently be out with the archive for long periods of time when they may be needed. Our own observations are echoed in a recent commentary on digital pathology experience from a Dutch group (14) which remarks in the context of preparation for an MDT meeting, that “*a resident would spend about a full day collecting slides from the archive or the desks of our 30 pathologists and residents*”.

Finally, we were also able to capture the complexities of the movement of personnel with the RFID badges worn by study participants, which varied according to their role (pathologist vs. biomedical scientist vs. secretary). There were clearly busy locations in terms of the workflow points, and it is predicted that a digital workflow would negate a substantial amount of this personnel movement, creating another time efficiency that may otherwise not have been so easily evidenced. In the current setting of the pandemic, this movement is not without potential risk, and the impact of DP in this context (29, 30), will likely continue into the foreseeable future.

On the basis of the literature to date, we would regard our study as probably the longest in terms of duration, and the largest in terms of the number of cases for which data has been captured, and with analysis of activity of the broadest range and number of personnel. We acknowledge that some data is incomplete but overall the data presented offers a detailed analysis of the “post-laboratory” journey of a surgical case; to the best of the authors knowledge no other studies to date have reported on continuous capture of similar data over such a time period. As we have shown in our study, the complexity of the analog pathway cannot be under-estimated, and efficiencies gained within a digital workflow will be multifactorial and not limited to saving time and potential improvements in turnaround times.

The potential inefficiencies seen in an analog workflow that could be addressed with digital pathology relate to batching with redundant time periods, physical movement of staff and cases, and loss or temporary unavailability of slides for which time is spent on locating them. Although concrete time efficiency savings are difficult to extrapolate in this study when moving from analog to digital, clear potential for improved patient safety aspects with DP are seen due to case traceability, and availability of slides for review and discussion at MDT, as well as potential for improvement in diagnostic reporting times resulting from more timely case availability.

Importantly, access to digital pathology underpins the potential benefit anticipated from the future integration of artificial intelligence into the workflow (31). It is challenging therefore to “pin down” exact cost savings that could be presented as part of a business case to support transition to DP as there are many “softer” savings and additional benefits that do not have direct cost implications. There is also the potential for revenue opportunities within the digital laboratory which may offset financial outlay in the longer term (6). Such opportunities may be within the context of increasing the caseload of a department enabled by digital working across sites, but also include contribution to more academic avenues such as image analysis and computational pathology. Indeed, in the UK there is an increasing recognition of the value of industry partnerships with the NHS and universities to aid the development of advanced diagnostics and ultimately improvement in patient care, which may also provide revenue back to the NHS.

## Study limitations

The main limitation of our study in terms of the impact on the transferability of the potential efficiency gains that we have reported, is that it has been conducted within a single institution. This is not unique in terms of pre-existing studies looking at efficiency related to analog and digital workflows, but transferability of the study outcomes to other laboratory settings will be dependent upon the workflow which, as we have discussed, is typically variable across laboratories. We have however highlighted the aspects of our study data that we feel will be most transferable, and aspects such as complexity of the pathways that cases follow in the analog setting is likely to be a consistent feature across institutions.

We have acknowledged within our Section Results the limitations of our study in terms of the deficiencies of data collected during study period 1 in particular, which was attributed to technical issues related to the placement of readers which were set up within a functioning laboratory setting. In this respect the study was ambitious in the aim of capturing data with readers necessarily set within fairly close confines in the workplace, but we wanted to be able to operate the study within a real-life setting with as little impact on the system being analyzed as possible. In spite of this relative limitation, we still captured the movement of over 1,000 cases, and had detailed data from 400 case journeys to allow meaningful analysis of the analog workflow. The complexity of the case journey was underestimated, and whilst this provides strong evidence for aspects of potential safety and quality gains with a digital workflow, it necessitated detailed manual downstream data analysis, with unavoidable redundancy of some data which had been collected. We recognize that we have not captured data from our LIMS which could have provided additional timestamps for analysis, such as those related to cases being booked out of the lab, or to reports being authorized by pathologists. We appreciate that this may have further augmented the study, but we feel that we have illustrated sufficient detail in the analog pathway to benefit the understanding of the pinch points in the workflow, and foresee that this will be beneficial even to the optimization of existing analog workflows in departments who are yet to consider the digital transition. Finally, whilst we have not covered the entire range of subspecialties within the department we do not believe this to impact upon the validity of the main take home messages which are related to complexity of pathways and the pinch points, which we feel will be similar regardless of the specialty analyzed. Certainly in our own department the workflow from lab to pathologist and pathologist back to the lab, would not generally be specialty-specific.

## Conclusion

In this study we have utilized a novel approach to capturing the glass slide-based (analog) cellular pathology workflow within a large teaching hospital setting, using RFID technology. The complexity of the workflow that we have illustrated is evidence of the challenge in capturing workflow data, which may in part explain the relative lack of evidence of efficiency savings by “going digital” and implementing digital pathology to date. We demonstrate the lag times in the analog workflow before a case reaches a pathologist for reporting, and the patterns of analog working that will be redundant with DP, and the potential for at least some unnecessary time in physical movement of slides and personnel around the department, and redundant time when cases are not moving through the workflow. Our intention is to reassess the workflow with DP fully implemented within our laboratory, however since the analog data was captured the unanticipated changes imposed on our working lives by the COVID-19 pandemic has meant that a future study will need to take into consideration the resultant change in work patterns, and significantly any remote working. Although we recognize that there may be differences in workflows between laboratories, this study is an attempt to provide evidence around potential efficiencies within this specific but significant portion of the workflow of a surgical case which may benefit a business case for DP in other settings. Importantly having detailed tracking data gives us a baseline position and enables the creation of complementary lean workflows for pathologists and lab staff to optimize chances of cases being available as soon as possible and navigating pinch points.

## Data availability statement

The raw data supporting the conclusions of this article will be made available by the authors, without undue reservation.

## Author contributions

LB, KW, DS, and CV contributed to the conceptualization of the study and the study design. Technical input in relation to the RFID equipment and set-up, and the data acquisition and initial processing was provided externally, as detailed within the manuscript. LB led the analysis of the data with input from KW and CV. The manuscript was drafted by LB and reviewed and revised by CV. All authors contributed to the final drafting of the manuscript and approved the final paper.

## Funding

This paper was supported by the PathLAKE Center of Excellence for Digital Pathology and Artificial Intelligence which was funded from the Data to Early Diagnosis and Precision Medicine strand of the HM Government's Industrial Strategy Challenge Fund, managed, and delivered by Innovate UK on behalf of UK Research and Innovation (UKRI). Grant Ref: File Ref 104689/Application Number 18181. CV and LB are part funded by the National Institute for Health Research (NIHR) Oxford Biomedical Research Center (BRC). CV also receives funding support from the Chinese Academy of Medical Sciences (CAMS) Innovation Fund for Medical Science (CIFMS), China (Grant Number: 2018-I2M-2-002). Philips (Amsterdam, Netherlands) provided the following funding for this study: the RFID equipment (Impinj, Seattle, WA, USA), data capture software (AUCXIS, Stekene, Belgium), and the preliminary data analysis which was commissioned from CQM (Consultants in Quantitative Medicine, Eindhoven, Netherlands).

## Conflict of interest

JR is a co-founder of Ground Truth Labs. CV is the principal investigator of a study evaluating Paige Prostate. University of Oxford and Oxford University Hospitals NHS Foundation Trust are part of the PathLAKE consortium. PathLAKE has received in kind industry investment from Philips. Philips (Amsterdam, Netherlands) provided the following funding for this study: the RFID equipment (Impinj, Seattle WA USA), data capture software (AUCXIS, Stekene Belgium), and the preliminary data analysis which was commissioned from CQM (Consultants in Quantitative Medicine, Eindhoven, Netherlands). RR has a role as Product Manager for Philips (Digital and Computational Pathology). The remaining authors declare that the research was conducted in the absence of any commercial or financial relationships that could be construed as a potential conflict of interest.

## Publisher's note

All claims expressed in this article are solely those of the authors and do not necessarily represent those of their affiliated organizations, or those of the publisher, the editors and the reviewers. Any product that may be evaluated in this article, or claim that may be made by its manufacturer, is not guaranteed or endorsed by the publisher.

## Author disclaimer

Views expressed are those of the authors and not necessarily those of the PathLAKE Consortium members, the NHS, Innovate UK or UKRI.
