# Supplementary material for: RFID analysis of the complexity of cellular pathology workflow—An opportunity for digital pathology
Source: Front Med (Lausanne). 2022 Aug 1;9:933933. doi: 10.3389/fmed.2022.933933 (PMC9377528; doi:10.3389/fmed.2022.933933)
Supplement: Supplementary file 1 [file Data_Sheet_1.PDF]

SUPPLEMENTARY DATA

Supplemental figure 1

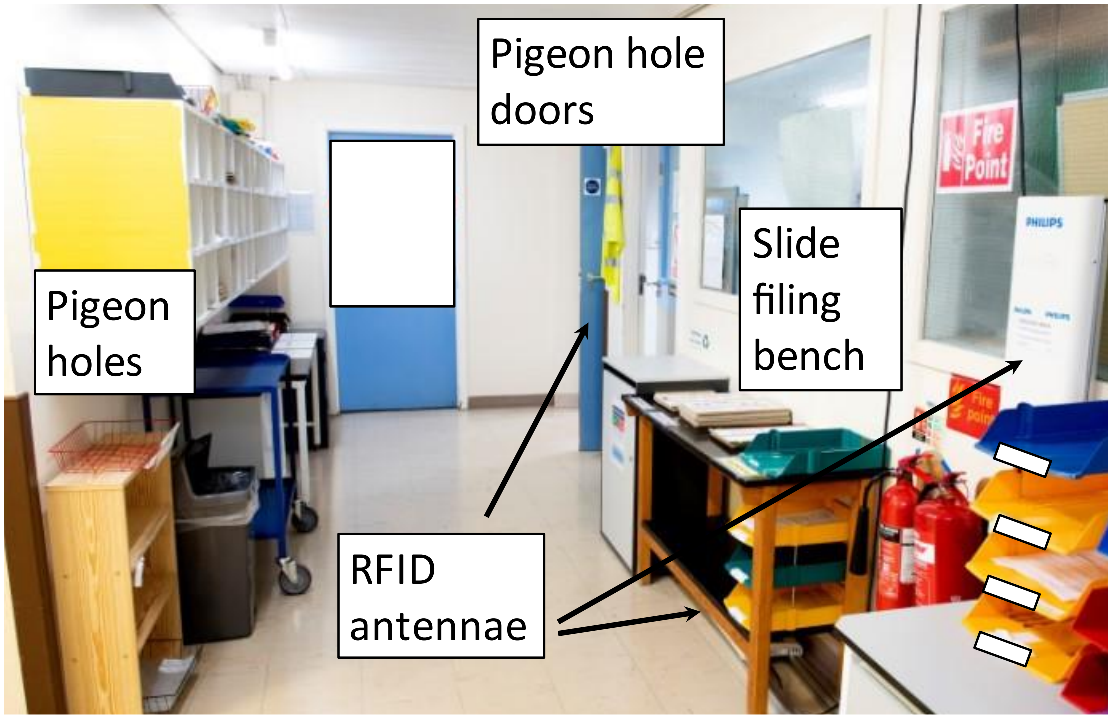

Positioning of the RFID readers was critical to ensure the tags were registered at the correct point in the workflow. This was challenging in the busy and relatively small space within the department where pathologists and other staff would collect slides (from the PH) and return the slides (filing bench), and the position was dictated by the distance the readers needed to be apart in order to track the tags accurately. This figure illustrates the challenge of positioning of the readers to track cases reaching the PH from the lab at the start of the workflow, and to keep this separate from the cases being returned to filing toward the end of the workflow. PH = pigeon holes.

Supplemental figure 2

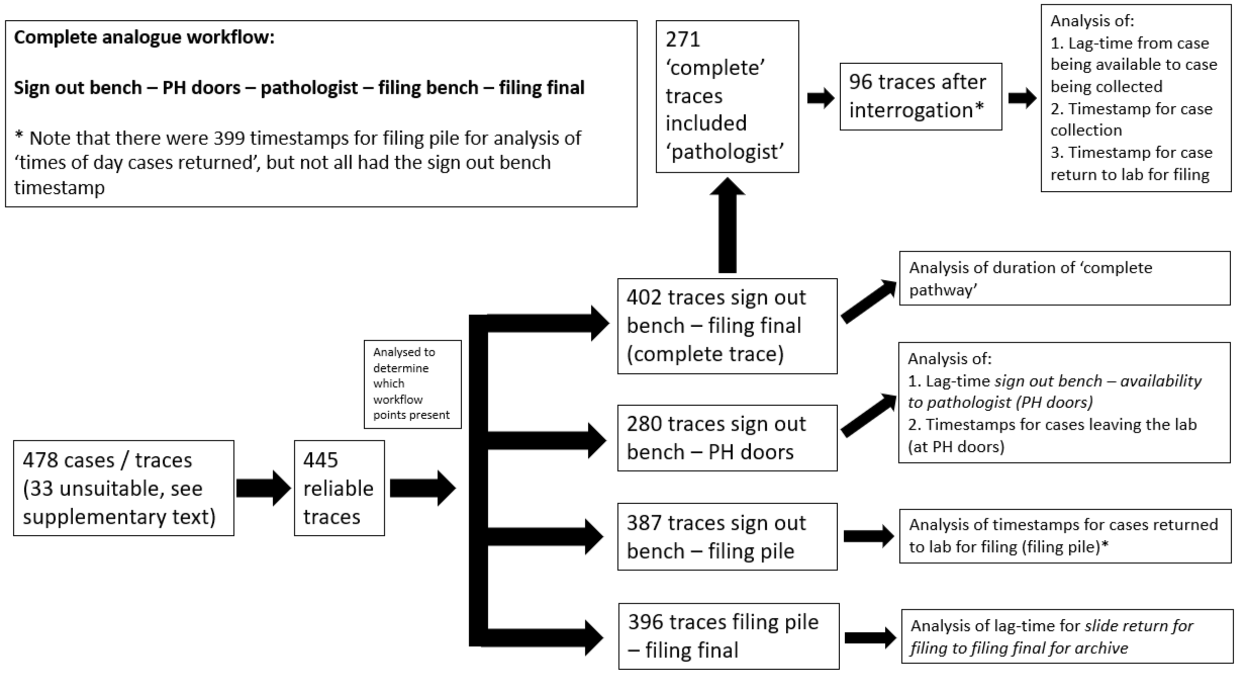

Trace data for study 2 is shown in detail. There were 478 cases/traces recorded during the study, however a timestamp had not been registered at each of the workflow points for all 478 traces. The traces were therefore analysed individually to determine which timestamps were available and to ensure that these were logical. At this point 33 traces were excluded. The flowchart illustrates the numbers of traces which included various combinations of timestamps, and what data was analysed from these traces. This data is presented in the main text (results). PH = pigeon hole.

Supplemental text

Study 2 Results – details pertaining to the analysis of traces

There were 478 traces captured for study 2, of which 33 were excluded following review of each of the individual traces to sense-check them; for example, for 14 cases the only timestamp was at the filing final, or sign out bench and filing pile (4 cases), or where the timestamps at workflow points of most interest were not available.

Analysis of the trace data remaining (445 traces) was in accordance with the available timestamps for that trace; some traces had more complete data than others and it was necessary to make an assessment of which trace patterns contributed to which of the data points being analysed (see also supplementary figure 2).

402 of the cases were traced from the sign out bench to the filing final bench in the lab (90% of those with a reliable trace), interpreted as a 'complete' trace. These traces provided data of the time for the complete journey of a case. 280 traces included timestamps for the sign out bench and pigeon hole (PH) doors, and could be used to capture lag time between a case being 'ready' in the lab and when it was actually made available to a pathologist (PH doors considered equivalent to placement in the pathologist PH). Timestamps at the PH doors also provided data to illustrate the pattern of physical activity in the workflow, in this instance a timestamp of when cases typically left the lab. 387 traces included sign out bench and filing file, and 396 traces included filing pile and filing final, the latter providing an indication of another aspect of potential inefficiencies of the analogue workflow whereby returned slides waited to be physically transported to the final filing point to be archived.

In addition, more detailed analysis of those traces which included a 'pathologist' timestamp allowed capture of important aspects of the analogue workflow; lag-time between a case being available at the PH for reporting and the time at which it was collected by a pathologist, and the timestamps to illustrate the pattern of pathologist activity in relation to collection of cases and return of cases to the lab for filing. Of the 402 complete traces, 271 traces included 'pathologist' within the journey. These were cases that had passed a reader placed at a physical workflow point regarded as being associated with relocation of a case to a pathologist's office (level 1 corridor or door to the 8th floor). Some included 'pathologist' on more than one timestamp, and these traces were further sense-checked to assess the logic of the timestamps at each of the designated workflow points. Any cases with an element of doubt or even minor anomalous datapoints were excluded from this more detailed analysis, leaving 96 case traces with a pathologist timestamp which were felt to be entirely robust.

Of the 96 'usable' traces which included a pathologist, there were 54 complete traces from the sign out bench – PH doors – pathologist – filing pile – filing final (pattern 1). There were 37 traces starting at the

sign out bench and then including pathologist, filing pile and filing final, but without a timestamp at the PH doors (pattern 2). There were a further 2 traces without ‘filing final’ but including PH doors and pathologist (pattern 3), and a further 2 that included PH doors and pathologist but without the filing pile prior to filing final (pattern 4). In spite of the lack of timestamps at one point in the journey for patterns 2 to 4, there was data from those traces which detailed either the time taken for a case to leave the lab and be collected by a pathologist (patterns 1, 3, 4) or the filing time of a case (patterns 1 and 2).
